# Supplementary material for: The interactome of intact mitochondria by cross-linking mass spectrometry provides evidence for coexisting respiratory supercomplexes
Source: Mol Cell Proteomics. 2017 Dec 8;17(2):216–32. doi: 10.1074/mcp.RA117.000470 (PMC5795388; doi:10.1074/mcp.RA117.000470)
Supplement: Supplemental Data [file supp_RA117.000470_133922_0_supp_23978_fzffwf.zip › spectra_annotation/mito_spectra_annotation/319-1-10-1-10-1.pdf]

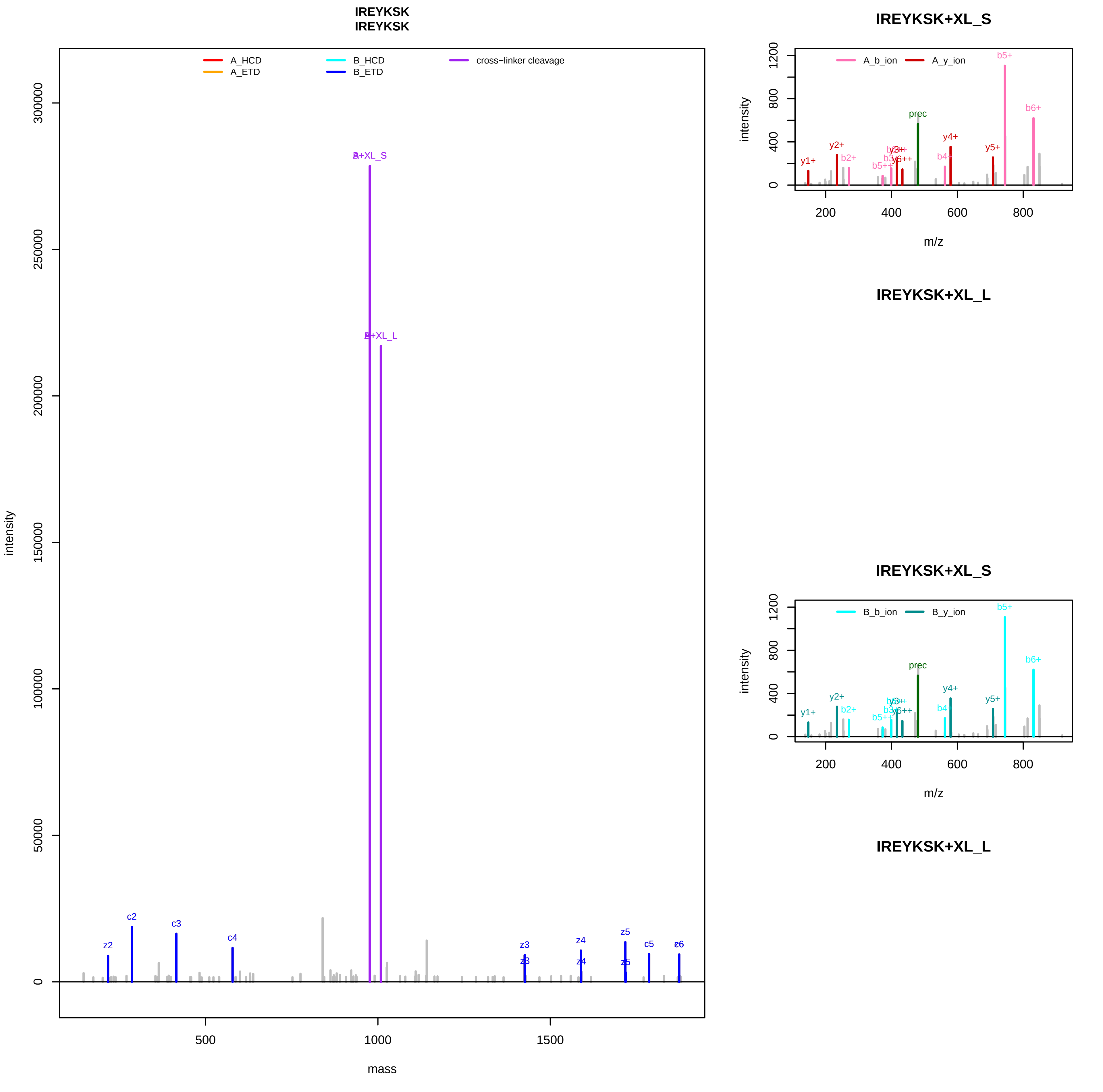
IREYKSK
IREYKSK
A_HCD
A_ETD
B_HCD
B_ETD
cross−linker cleavage
intensity
mass
300000
250000
200000
150000
100000
50000
0
500
1000
1500
B+XL_S
B+XL_L
z2
c2
c3
c4
z3
z4
z5
c5
c6
IREYKSK+XL_S
A_b_ion
A_y_ion
prec
y1+
y2+
b2+
b5++
y3+
y6++
y4+
b4+
y5+
b5+
b6+
intensity
m/z
1200
800
400
200
600
IREYKSK+XL_L
IREYKSK+XL_S
B_b_ion
B_y_ion
IREYKSK+XL_L
